# Dietary adherence and cognitive performance in older adults: insights from a nationwide survey in China

**DOI:** 10.3389/fnut.2025.1605016

**Published:** 2025-07-30

**Authors:** Yameng Fan, Yan Yu, Jiao Tan, Yonghong Ma, Ke Men

**Affiliations:** ^1^School of Public Health, Xi’an Medical University, Xi’an, China; ^2^School of Public Health, Xi’an Jiaotong University, Xi’an, China

**Keywords:** dietary guidance, CHEI, cognitive performance, older adults, CHNS

## Abstract

**Background:**

Dietary guidelines, integrating the latest scientific evidence with traditional dietary culture, are regularly issued to provide dietary recommendations and promote health. However, the relationship between adherence to dietary guidelines and cognitive health has yet to be fully elucidated. In this study, we specifically examine the association between adherence to the Dietary Guidelines for Chinese (DGC) and cognitive performance among older adults.

**Methods:**

We analyzed data from the China Health and Nutrition Survey (CHNS) (2011 and 2015), including participants aged ≥60 years. Adherence to the DGC was assessed using the Chinese Healthy Eating Index (CHEI). Cognitive performance was evaluated across the following three domains: (1) memory (immediate and delayed recall), (2) attention/executive function (counting backward from 20), and (3) processing speed (serial 7 subtraction). A composite *z*-score was derived to represent global cognition. Multivariate logistic regression and restricted cubic spline models were used to assess associations.

**Results:**

Among 2,174 older adults, higher CHEI scores were significantly associated with better performance at counting backward (attention/executive function, *p* < 0.05), serial 7 subtraction (processing speed, *p* < 0.01), and global cognition (*p* < 0.01). A significant interaction effect was observed between the CHEI and nationality in relation to cognitive performance (*p*_interaction_ <0.05). Among the 17 CHEI components, only adherence to the recommended intake of fish and seafood (OR: 0.55; 95% CI: 0.37, 0.82) was independently linked to better cognitive performance. Sensitivity analyses excluding individuals with diabetes or overweight or obesity, and treating cognitive performance as a continuous outcome yielded consistent results (*p* < 0.05).

**Conclusion:**

Greater adherence to the DGC was associated with better cognitive performance, particularly in processing speed and attention/executive function. Promoting adherence to dietary guidelines may be a viable strategy for preserving cognitive health in aging populations.

## Introduction

With the advances in modern medicine and economy, population aging has become a global phenomenon. According to its seventh population census conducted in 2020, China has 264 million people aged 60 years and above, accounting for 18.7 percent of its population of 1.4 billion (1). In this context, age-related degeneration, especially cognitive decline among the elderly, has become a major public health concern.

To an extent, cognitive decline heralds the development of mild cognitive impairment (MCI) and different types of dementia. Although the occurrence of dementia is closely related to age, it is not an inevitable consequence of aging. Around 40% of worldwide dementia could theoretically be prevented or delayed based on the 2020 report of the Lancet Commission (2). Accordingly, it is imperative to identify the potentially modifiable risk factors to promote cognitive health.

Diet, as a modifiable factor, is closely related to daily life. Accumulating evidence suggests that individual foods or nutrients, such as fruit, fish, vitamins and polyunsaturated fatty acids, are associated with cognitive performance among the elderly (3–6). Considering the cooperation and antagonism between nutrients and foods, contemporary research examining the link between diet and cognition has gradually transitioned into assessments of overall diet quality.

Diet quality is typically assessed using either guideline-based scoring systems [e.g., Chinese Dietary Guidelines Index (CDGI), Chinese Diet Quality Index (CDQI), and Chinese Healthy Eating Index (CHEI)] or pattern-based indices [e.g., Mediterranean Diet (MED) and Dietary Approaches to Stop Hypertension (DASH)]. Previous studies have primarily focused on diet quality assessed by the MED pattern, but this dietary pattern may not be appropriate for non-Mediterranean countries, such as the U.S., Denmark, and China (7). Investigating the associations between adherence to national dietary guidelines and cognitive performance in local populations holds greater practical significance. However, such research remains relatively limited and controversial (8, 9). Compared to existing Chinese diet quality indices, the CHEI offers three distinctive advantages: (1) it incorporates a more comprehensive range of food components, (2) it is designed as a continuous scoring system, and (3) it is the first index to quantify food consumption using standardized portion sizes as recommended in the DGC. These features enhance its validity for assessing dietary quality in Chinese populations.

Based on the data from the China Health and Nutrition Survey (CHNS) for 2011 and 2015, we examined whether alignment with the Dietary Guidelines for Chinese (DGC) measured using the CHEI, was related to cognitive performance among the Chinese elderly.

## Methods

### Study population

The data used in this study were publicly available from the CHNS,^1^ which is an ongoing cohort study that was initiated in 1989 and followed up in 1991, 1993, 1997, 2000, 2004, 2006, 2009, 2011, and 2015 (the latest open-access date). The methodological framework and operational protocols of the CHNS have been extensively documented (10). Briefly, the CHNS employs a multistage random cluster sampling approach to select representative individuals from 15 provinces (municipalities or autonomous regions) across China. A primary objective of the CHNS is to assess the nutritional, socioeconomic, and health status of the Chinese population.

**Figure 1:**
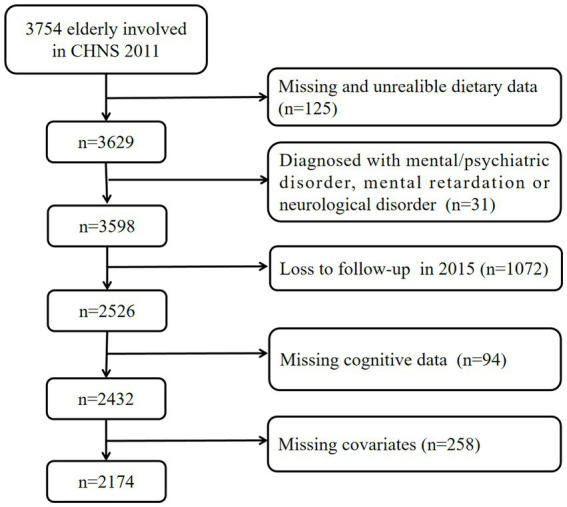
Flow diagram of the study sample selection.

Our analysis used data from the two most recent available waves (2011 and 2015) to explore the associations between adherence to the DGC and cognitive performance. Dietary and baseline information was collected from the 2011 wave (as a baseline), and cognitive performance was assessed using the 2015 wave. The following exclusions were applied to the 3,754 subjects aged 60 years or older at the baseline: (1) those with missing dietary data or extreme energy intake (<800 or >6,000 kcal/day for males and <600 or >4,000 kcal/day for females); (2) those with mental/psychiatric disorders, intellectual disability, or neurological disorders; (3) those lost to follow-up in the 2015 wave; (4) those with missing cognitive data; and (5) those with missing covariate data. Subsequently, a total of 2,174 participants were included in the final analysis (Figure 1). As shown in Supplementary Table S1, participants completing follow-up were significantly younger (67.8 vs. 70.9 years), more likely to live in rural site, less likely to have diabetes, and had higher energy intake compared to those lost to follow-up. No other significant differences were observed between the groups.

### Dietary assessment and the calculation of the CHEI

Dietary intake was assessed using three consecutive 24-h dietary recalls at the individual level and a food inventory weighing method at the household level. Daily energy and nutrient intakes were estimated using the China food composition databases (2002 and 2004 versions). Proportions of condiments (such as cooking oils, sodium, and added sugars) at the household level were allocated to individuals based on their calculated energy intake at the individual level.

The CHEI was developed and validated by Yuan et al. (11, 12) to assess adherence to the DGC. The CHEI comprises 17 components (food groups or nutrients), including 12 adequacy components (total grains, whole grains and mixed beans, tubers, total vegetables, dark vegetables, fruit, dairy, soybeans, fish and seafood, poultry, eggs, and seeds and nuts) and 5 moderation components (red meat, cooking oil, sodium, added sugar, and alcohol). After converting gram values to standard portion (SP) units, the scoring of these components was calculated per 1,000 kcal of energy density, with the exception of alcohol (expressed as an absolute weight) and added sugars (expressed as a percentage of energy). The detailed scoring criteria for individual components are presented in Supplementary Table S2. Individual component scores were independently calculated and aggregated to generate a composite score, which ranged from 0 to 100 points. Elevated scores reflected stronger adherence to the DGC.

### Assessment of cognitive performance

A subset of items from the modified Telephone Interview for Cognitive Status (TICS-m) was administered to individuals in the CHNS who were 55 years of age or older. These items included immediate and delayed recall of a 10-word list (20 points), counting backward from 20 (2 points), and serial 7 subtraction (5 points).

1. Immediate and delayed recall of a 10-word list: This item was used to evaluate the cognitive subdomain of memory. Participants were asked to memorize 10 unrelated words within 2 min for the immediate recall test and to recall these words at the end of the session for the delayed recall test. The score was based on the total number of correctly recalled words.
2. Counting backward from 20: This item was used to assess the cognitive subdomain of attention/executive function. Participants were asked to count backward from 20 to 1. Scores were assigned as follows: 2 points for a correct response on the first attempt, 1 point for a correct response on the second attempt, and 0 points for incorrect responses on both attempts.
3. Serial 7 subtraction: This item was designed to evaluate the cognitive subdomain of processing speed. Participants were requested to execute five successive subtractions of 7 starting from 100. One point was awarded for each correct subtraction.

Global cognitive performance was calculated using aggregating *z*-scores [(test score − mean score)/standard deviation (SD)] for the above items. Elevated values for all items corresponded with superior cognitive performance. The lowest quartile of *z*-scores based on age group (60–69, ≥70) was defined as poor cognitive performance, which was consistent with previously used methods (13, 14).

### Covariates

Sociodemographic variables such as age, gender (male and female), nationality (Han and minority), location (urban site and rural site), education level (middle school and below, and high school and above), and income (inflation-adjusted household income per capita, categorized into tertiles as low, middle, and high) were collected.

Lifestyle variables included participants who had smoked at least one cigarette per day, who were defined as smokers, and participants who had consumed alcohol in the past year, who were defined as drinkers. Leisure sedentary time was assessed based on the total time spent sitting in a week (excluding time spent sleeping).

Body measurements, were obtained, including weight (kg) and standing height (cm). Body mass index (BMI) was calculated as weight divided by the square of height.

Following a 10-min seated rest period, blood pressure was measured according to standardized protocols. Three sequential readings were recorded, with the mean systolic (SBP) and diastolic (DBP) blood pressure values used for the analysis. Hypertension was operationally defined as follows: (1) average SBP ≥140 mmHg, (2) average DBP ≥90 mmHg, or (3) self-reported use of physician-prescribed antihypertensive medications. Diabetes was defined based on a physician’s diagnosis (self-reported).

### Statistical analysis

The characteristics of the study population were described as the mean ± SD for continuous variables and the number (percentage) for categorical variables. The CHEI was categorized into quartiles (quartile 1: <25th percentile; quartile 2: ≥25 to 50th percentiles; quartile 3: ≥50 to 75th percentiles; and quartile 4: ≥75th percentiles). Differences in the characteristics between groups were tested using a one-way analysis of variance for continuous variables and a chi-square test for categorical variables. The relationships between the CHEI and cognitive performance were evaluated using binary logistic regression models. We considered the median CHEI value within each quartile to be a continuous variable to examine the trends associated with ascending quartiles of the CHEI. In Model 1, adjustments were made for age, gender, and nationality. In Model 2, additional adjustments were incorporated for location, energy intake, drinking status, smoking status, and leisure sedentary time. Model 3 was further adjusted for education, income, BMI, hypertension, and diabetes. Restricted cubic splines with knots at the 5th, 50th, and 95th percentiles of the exposure distribution were applied to examine to explore the dose–response relationships between the CHEI (as a continuous variable) and poor cognitive performance based on the SAS macro “*%RCS_Reg*” (15). Sensitivity analyses were performed by excluding participants with (1) diabetes or (2) overweight or obesity, and (3) treating cognitive performance as a continuous outcome. Furthermore, we performed stratified analyses to determine if these associations varied according to different factors, such as age group (60–69 and ≥70), gender (male and female), or nationality (Han and other minorities). For exploratory purposes, we investigated the relationship between CHEI components (dichotomized as meeting or not meeting the DGC) and poor cognitive performance to determine if statistically significant associations exited that could be ascribed to particular components. STATA version 16.0 (StataCorp, College Station, TX, United States) was employed for data management and the main analyses. SAS version 9.4 (SAS Institute, Cary, NC, United States) was used for the dose–response curve analyses. A two-tailed *p*-value <0.05 was considered to be statistically significant.

## Results

### Descriptive statistics

The baseline characteristics of participants across the CHEI quartiles are shown in Table 1. Participants with a higher CHEI were more likely to be Han and non-smokers; live in urban site; have diabetes; have a higher BMI, education level, and income; and have increased leisure sedentary time (*p* < 0.05). We did not observe any significant disparities for age, gender, energy intake, drinking status, or hypertension across the quartiles of the CHEI.

**Table 1:** Baseline characteristics of the overall target population according to CHEI quartiles^a^.

Characteristics	CHEI				
	Q1^b^	Q2	Q3	Q4	*p* ^c^
*n*	544	543	544	543	
Age (years)-baseline	68.1 ± 6.3	67.6 ± 6.3	67.6 ± 6.3	67.4 ± 6.3	0.389
Age (years)-2015	72.2 ± 6.3	71.7 ± 6.3	71.7 ± 6.3	71.5 ± 6.3	0.318
Gender, *n* (%)					0.185
Male	239 (43.9)	249 (45.9)	245 (45.0)	216 (39.8)	
Female	305 (56.1)	294 (54.1)	299 (55.0)	327 (60.2)	
Nationality, *n* (%)					<0.001
Han	455 (83.6)	471 (86.7)	479 (88.1)	507 (93.4)	
Others	89 (16.4)	72 (13.3)	65 (11.9)	36 (6.6)	
Location, *n* (%)					<0.001
Urban site	133 (24.5)	166 (30.6)	205 (37.7)	333 (61.3)	
Rural site	411 (75.5)	377 (69.4)	339 (62.3)	210 (38.7)	
Education level, *n* (%)					<0.001
Middle school and below	503 (92.5)	485 (89.3)	456 (83.8)	374 (68.9)	
High school and above	41 (7.5)	58 (10.7)	88 (16.2)	169 (31.1)	
Income, *n* (%)					<0.001
Low	253 (46.5)	207 (38.1)	172 (31.6)	93 (17.1)	
Moderate	165 (30.3)	215 (39.6)	193 (35.5)	153 (28.2)	
High	126 (23.2)	121 (22.3)	179 (32.9)	297 (54.7)	
Energy intake (kcal/day)	1839.9 ± 708.3	1865.4 ± 644.9	1863.7 ± 625.1	1829.7 ± 626.7	0.753
Sedentary time (hour/week)	16.4 ± 14.8	17.5 ± 16.5	17.2 ± 11.6	23.2 ± 14.9	<0.001
Smoker, *n* (%)					<0.001
Yes	163 (30.0)	161 (29.6)	123 (22.6)	110 (20.3)	
No	381 (70.0)	382 (70.3)	421 (77.4)	433 (79.7)	
Drinker, *n* (%)					0.374
Yes	134 (24.6)	150 (27.6)	153 (28.1)	133 (24.5)	
No	410 (75.4)	393 (72.4)	391 (71.9)	410 (75.5)	
Body mass index (kg/m^2^)	23.2 ± 3.6	23.3 ± 3.3	23.7 ± 3.5	24.4 ± 3.4	<0.001
Hypertension, *n* (%)					0.991
Yes	263 (48.3)	262 (48.2)	267 (49.1)	262 (48.2)	
No	281 (51.7)	281 (51.8)	277 (50.9)	281 (51.8)	
Diabetes, *n* (%)					0.020
Yes	32 (5.9)	25 (4.6)	42 (7.7)	49 (9.0)	
No	512 (94.1)	518 (95.4)	502 (92.3)	494 (91.0)	

a Values are number (%) or means ± SD; CHEI, Chinese Healthy Eating Index.

b Q, Quartiles; Q1 represents the unhealthiest diet quality, Q4 represents the healthiest diet quality.

c *p*-values were calculated using chi-square tests for categorical variables and *F*-tests for continuous variables.

### CHEI and poor cognitive performance

As shown in Table 2, the highest CHEI quartile in Model 1 was significantly linked to decreased odds of poor cognitive performance (OR: 0.38; 95% CI: 0.27, 0.53; *p* < 0.001) compared to the lowest CHEI quartile. The relationship between the CHEI and poor cognitive performance was attenuated, but remained significant, after additional adjustments in Model 2 (OR: 0.48; 95% CI: 0.34, 0.68; *p* < 0.001) and Model 3 (OR: 0.60; 95% CI: 0.42, 0.85; *p* < 0.01). A significant linear trend was observed, indicating that the odds of poor cognitive performance significantly decreased with an increase in CHEI quartiles (*p*_trend_ <0.01). A continuous variable analysis revealed that a 1-SD increment in the CHEI was significantly linked to decreased odds of poor cognitive performance (*p* < 0.01).

**Table 2:** Odds ratios and 95% confidence intervals of the CHEI for poor performance^a^.

Models	CHEI					
	Q1^b^	Q2	Q3	Q4	*p*_trend_ ^c^	1 SD increase
Model 1^d^	1 (Reference)	0.81 (0.61, 1.08)	0.67 (0.50, 0.90)^**^	0.38 (0.27, 0.53)^***^	<0.001	0.71 (0.63, 0.79)^***^
Model 2	1 (Reference)	0.84 (0.63, 1.12)	0.71 (0.53, 0.96)^*^	0.48 (0.34, 0.68)^***^	<0.001	0.77 (0.68, 0.87)^***^
Model 3	1 (Reference)	0.84 (0.63, 1.13)	0.78 (0.57, 1.05)	0.60 (0.42, 0.85)^**^	<0.01	0.84 (0.74, 0.95)^**^

a CHEI, Chinese Healthy Eating Index; ^***^p < 0.001, ^**^p < 0.01 and ^*^p < 0.05.

b Q: Quartile, Q1 represents the unhealthiest diet quality, Q4 represents the healthiest diet quality.

c *p*_trend_: Test for trend based on a variable containing the median value for each quartile.

d Model 1: adjusted for age, gender, and nationality; Model 2: Model 1 + location, energy intake, drinking status, smoking status, and leisure sedentary time; Model 3: Model 2 + education, income, BMI, hypertension, and diabetes.

**Figure 2:**
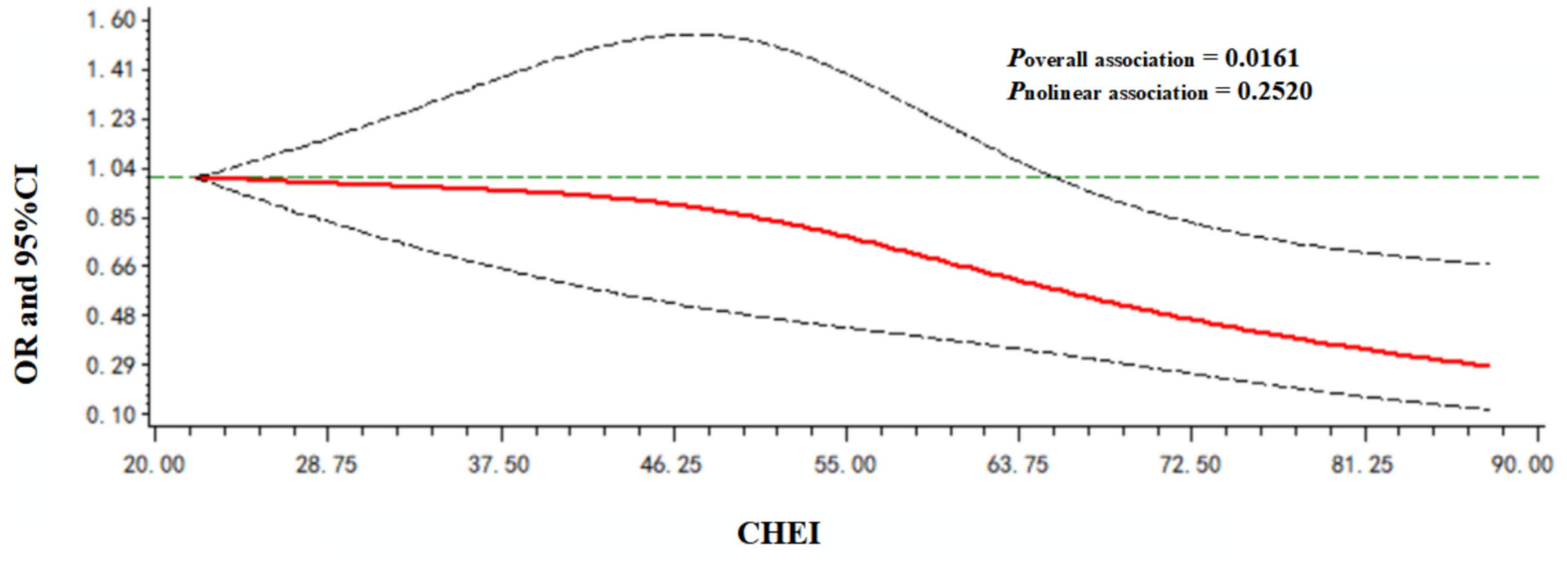
Dose–response relationship between the CHEI and poor cognitive performance. Models were adjusted for age, gender, nationality, location, energy intake, drinking status, smoking status, leisure sedentary time, education level, income, BMI, hypertension, and diabetes.

A dose–response relationship between the CHEI and poor cognitive performance was observed. As shown in Figure 2, the CHEI was negatively associated with poor cognitive performance in a linear manner (*p*_overall association_ = 0.0161; *p*_nonlinear association_ = 0.2520).

### CHEI and different cognitive subdomains

Table 3 displays the associations between the CHEI and poor cognitive performance for different subdomains. Higher CHEI was associated with significantly lower fully adjusted odds of poor cognitive performance in the counting backward from 20 test (*p*_trend_ <0.05) and the serial 7 subtraction test (*p*_trend_ <0.01). No significant association was observed between the CHEI and immediate and delayed recall.

**Table 3:** Odds ratios and 95% confidence intervals of the CHEI for poor performance in different cognitive domains^a^.

Cognitive domains	CHEI					
	Q1^b^	Q2	Q3	Q4	*p*_trend_ ^c^	1 SD increase
Memory						
Model 1^d^	1 (Reference)	0.74 (0.55, 1.01)	0.88 (0.65, 1.18)	0.82 (0.61, 1.12)	0.366	0.97 (0.87, 1.08)
Model 2	1 (Reference)	0.73 (0.54, 1.00)	0.85 (0.63, 1.16)	0.79 (0.58, 1.10)	0.259	0.96 (0.85, 1.07)
Model 3	1 (Reference)	0.73 (0.54, 1.00)	0.87 (0.64, 1.19)	0.84 (0.60, 1.16)	0.428	0.98 (0.87, 1.10)
Counting backward						
Model 1	1 (Reference)	0.88 (0.65, 1.19)	0.60 (0.43, 0.82)^**^	0.39 (0.28, 0.56)^***^	<0.001	0.69 (0.61, 0.79)^***^
Model 2	1 (Reference)	0.91 (0.67, 1.24)	0.64 (0.46, 0.89)^**^	0.54 (0.37, 0.79)^**^	<0.001	0.77 (0.68, 0.88)^***^
Model 3	1 (Reference)	0.92 (0.67, 1.25)	0.70 (0.50, 0.99)^*^	0.69 (0.47, 1.02)	<0.05	0.85 (0.74, 0.98)^*^
Subtraction						
Model 1	1 (Reference)	0.91 (0.67, 1.23)	0.69 (0.50, 0.94)^*^	0.35 (0.24, 0.50)^***^	<0.001	0.68 (0.60, 0.77)^***^
Model 2	1 (Reference)	0.93 (0.69, 1.26)	0.73 (0.53, 1.00)	0.44 (0.30, 0.64)^***^	<0.001	0.74 (0.65, 0.84)^***^
Model 3	1 (Reference)	0.94 (0.69, 1.27)	0.79 (0.57, 1.08)	0.51 (0.35, 0.75)^**^	<0.01	0.79 (0.69, 0.90)^***^

a CHEI, Chinese Healthy Eating Index; ^***^p < 0.001, ^**^p < 0.01, and ^*^p < 0.05.

b Q: Quartile, Q1 represents the unhealthiest diet quality, Q4 represents the healthiest diet quality.

c *p*_trend_: Test for trend based on a variable containing the median value for each quartile.

d Model 1: adjusted for age, gender and nationality; Model 2: Model 1 + location, energy intake, drinking status, smoking status, and leisure sedentary time; Model 3: Model 2 + education, income, BMI, hypertension, and diabetes.

### Interaction effects

**Figure 3:**
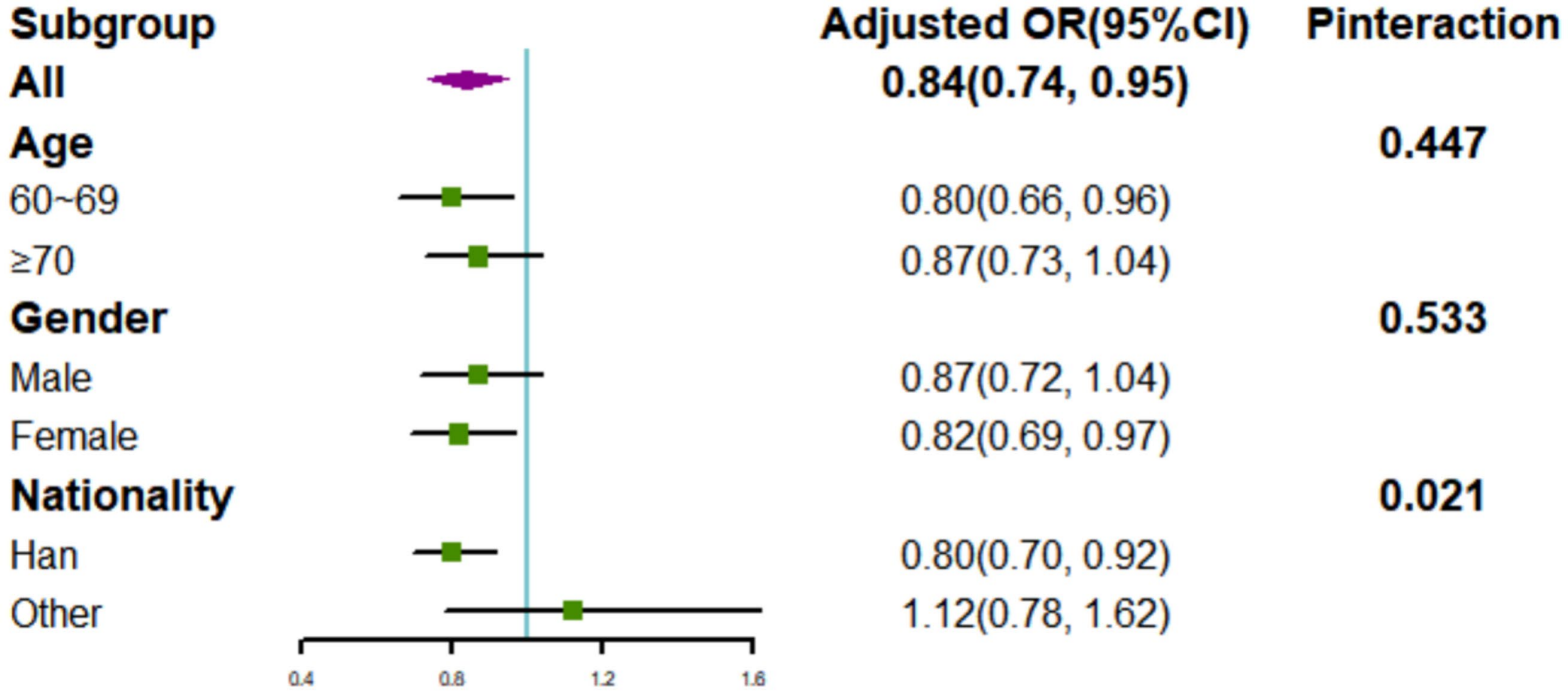
Associations of 1 SD increase in the CHEI with poor cognitive performance stratified by demographic characteristics (age, gender, and nationality). Models were adjusted for age, gender (except for gender subgroup analysis), nationality (except for nationality subgroup analysis), location, energy intake, drinking status, smoking status, leisure sedentary time, education level, income, BMI, hypertension, and diabetes.

As shown in Figure 3, a potential interaction effect was identified between the CHEI and nationality in relation to poor cognitive performance (*p*_interaction_ = 0.021). In the fully adjusted model, each 1-SD increment of the CHEI was linked to decreased odds of poor cognitive performance among the Han population (OR: 0.80; 95% CI: 0.70, 0.92), but not among other minority populations (OR: 1.12; 95% CI: 0.78, 1.62). No significant interaction effects were observed between the CHEI and age or gender (*p*_interaction_ >0.05).

### CHEI components and poor cognitive performance

**Figure 4:**
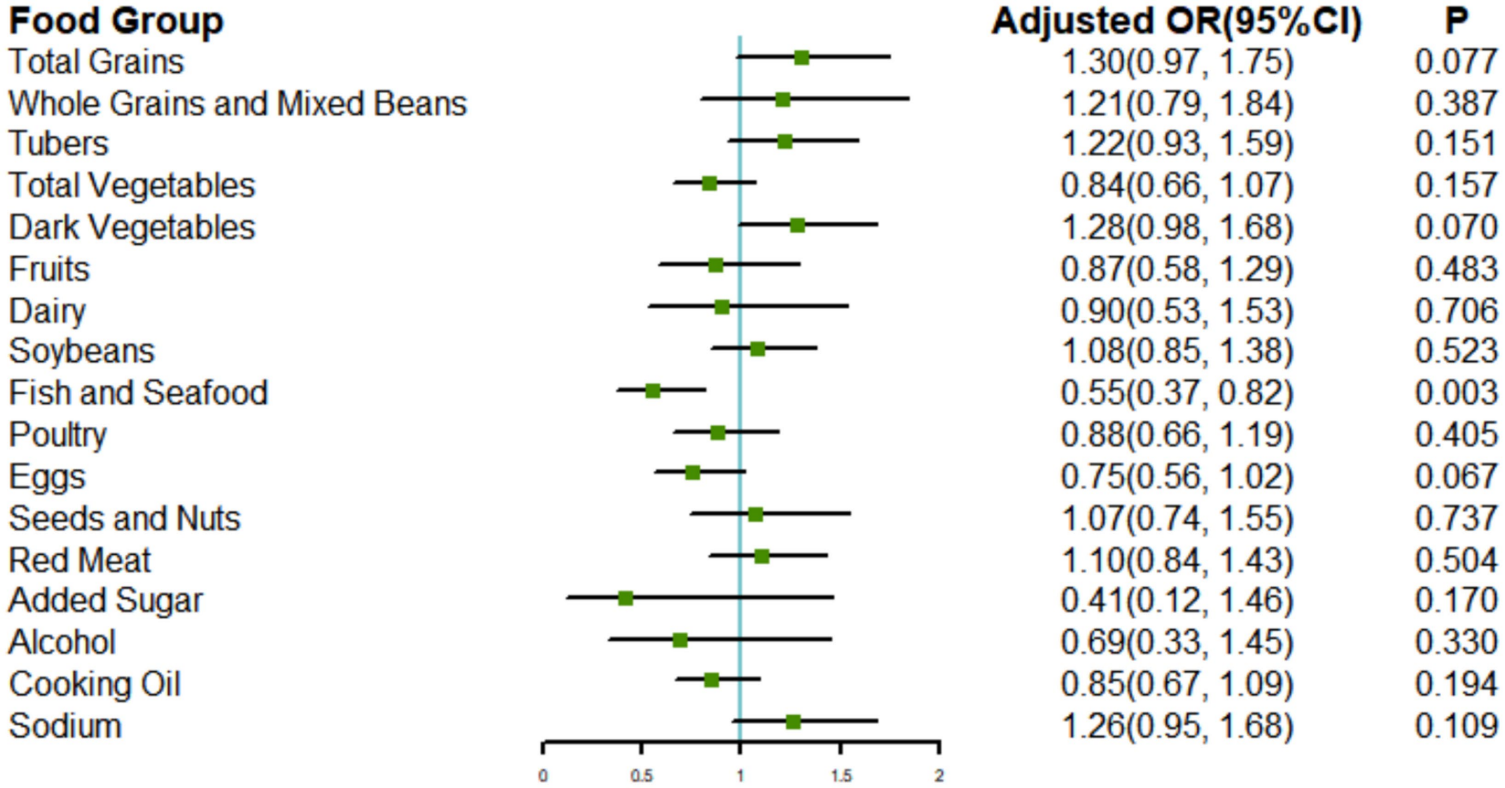
Associations of the CHEI components with poor cognitive performance. Models were adjusted for age, gender, energy intake, nationality, location, drinking status, smoking status, leisure sedentary time, education level, income, BMI, hypertension, diabetes, and other components.

Given the significant associations between the CHEI and poor cognitive performance, we conducted an exploratory analysis of the relationships between each CHEI component and poor cognitive performance. We adjusted for all covariates that were part of Model 3, along with other components. Of the 17 components, only adherence to the recommended consumption of fish and seafood was significantly associated with better cognitive performance (OR: 0.55; 95% CI: 0.37, 0.82) (Figure 4).

### Sensitivity analyses

The association between the CHEI and poor cognitive performance did not appreciably change in the sensitivity analyses after excluding participants who suffered from diabetes (*n* = 2,026; Supplementary Table S3) or overweight or obesity (*n* = 1,200; Supplementary Table S4). When treating cognitive performance as a continuous outcome (using composite *z*-scores), the association remained statistically significant in both linear regression (Supplementary Table S5) and dose–response analyses (Supplementary Figure S1), consistent with our primary categorical analysis.

## Discussion

In this study, we observed that compliance with the DGC, assessed using the CHEI, was associated with better cognitive performance among older Chinese adults (except for the memory subdomain). A negative relationship between the CHEI and poor cognitive performance was only observed among the Han nationality. Exploratory analyses based on CHEI components revealed a significant association between adherence to the recommended consumption levels of fish and seafood and superior cognitive performance, which may be attributed to the rich content of omega-3 polyunsaturated fatty acids in fish and seafood, especially docosahexaenoic acid (DHA).

Previous studies have explored the relationship between diet quality and cognitive health among older adults, but most of them have focused on diet quality assessed using the MED pattern (16, 17). The traditional Chinese dietary pattern differs from the MED pattern in terms of the food composition and cooking methods. The diet-quality scoring system based on the MED pattern only considers the relative intake levels of food or nutrients (below or above the median of the target population) rather than the actual intake levels, which may limit the practical guiding significance. In this study, we evaluated diet quality based on national dietary guidelines to more accurately reflect the real eating habits of the local population (e.g., food preferences, cooking methods, etc.). In addition, several cognition-specific indices, such as the Mediterranean-Dietary Approaches to Stop Hypertension Intervention for Neurodegenerative Delay (MIND) diet and the Oriental Intervention for Enhanced Neurocognitive Health (ORIENT) diet, have been developed and shown to exert significant neuroprotective effects (18, 19). Unlike these dietary patterns, which primarily emphasize neuroprotective foods, the CHEI provides a more comprehensive evaluation of overall dietary quality by assessing 17 components—including both adequacy and moderation metrics—thereby facilitating the capture of multiple health outcomes beyond cognition. More importantly, the CHEI’s alignment with China’s national dietary recommendations enhances its public health relevance, making it easier to translate into clinical and policy interventions for the Chinese population. Future studies incorporating both guideline-based and cognition-specific indices may further clarify their complementary roles across different populations.

Research on diet quality based on national dietary guidelines and associations with cognitive outcomes is relatively limited and has primarily been conducted in Western countries. Adherence to dietary guidelines has been associated with better cognitive performance, both cross-sectionally (in the Australia and U.S.) (9, 20) and longitudinally (over 15 years in the Netherlands and 11 years in the U.S.) (21, 22). To the best of our knowledge, there are two studies exploring the association between CHEI and cognitive health among the Chinese population. In the Chinese Longitudinal Healthy Longevity Study (CLHLS), a focused analysis of the mediating role of psychological balance and depressive symptoms between CHEI and cognitive function among rural older adults was conducted (23). Compared with this study, our research specifically enrolled older adults from both urban and rural sites. We conducted a comprehensive investigation of the relationship between the CHEI and cognitive function, including: (1) main association analysis, (2) cognitive subdomain analysis, (3) CHEI components-specific analysis, (4) stratified analysis, and (5) sensitivity analysis. Another study from CHNS (2004 & 2006) suggested that better diet quality assessed by CHEI improves cognitive performance in Chinese adults aged 55+ (24). Compared with this earlier work, our research possesses four key methodological improvements: (1) utilization of the latest open-access CHNS data (2011 & 2015) focusing on Chinese older adults aged ≥60 years; (2) standardized cognitive assessment using *z*-scores rather than raw scores; (3) comprehensive adjustment for potential confounders, including ethnicity and energy intake; and (4) rigorous additional analyses examining both cognitive subdomains and CHEI dietary components. Most notably, we made three novel discoveries: first, adherence to the DGC showed domain-specific cognitive benefits. Second, the Han population exhibited significant positive associations that were not observed in ethnic minorities. Third, component-level analysis identified fish and seafood intake as the primary driver of these associations.

We observed that adherence to the DGC was related to processing speed and attention/executive function cognitive subdomains, but not to memory. Surprisingly, our previous study based on National Health and Nutrition Examination Survey (NHANES) data also discovered that compliance with the 2015–2020 Dietary Guidelines for Americans (DGA), as evaluated using the Healthy Eating Index-2015 (HEI-2015), was linked to better processing speed and executive function, but no associations were observed for memory in U.S. adults aged 60 years and above (25). Another study involving 819 older adults in Australia indicated that adherence to the MED was only related to better visuospatial cognition and not to any other cognitive subdomains (26). These findings indicate that particular healthy eating patterns might only confer advantages to certain specific subdomains of cognition. Future studies should incorporate multiple cognitive tests to comprehensively and thoroughly assess cognitive performance.

Our results are distinctively demonstrated that compliance with the DGC was positively correlated with cognitive performance for the Han nationality, but not for other minorities. Although existing research has yet to fully clarify why the link between diet and cognitive function varies across nationalities, potential factors leading to such national disparities may include dietary habits, food availability, education levels, traditional culture, and media influence. Previous studies have reported that meal behavior and dietary intake differed greatly in different Chinese nationalities (27, 28). Moreover, research has shown that the apolipoprotein E (APOE) ε2 allele exhibits a protective effect against cognitive impairment exclusively in the Han, but not in the Hui—the largest minority population in China—suggesting distinct nationality-specific risks for cognitive impairment (29). Another contributing factor could be insufficient sample sizes in minority group analyses, reducing statistical reliability. For example, a protective effect of adherence to the MED on cognition was only observed in non-Hispanic White Americans, who had a larger sample size, but not in minority groups with smaller sample sizes, such as non-Hispanic Black, Hispanic/Latino, or Asian Americans (30). Conversely, in a study with a large sample of Hispanic/Latino Americans, significant associations between diet and cognition were observed (31). Overall, different diet strategies might thus be emphasized for different Chinese nationalities.

Better cognitive performance was observed in those who aligned with the recommended intake of fish and seafood. A meta-analysis incorporating 21 prospective cohorts revealed that each additional weekly serving of fish (approximately 105 g) was associated with a 5% reduction in the risk of dementia (32). Findings from two large prospective cohort studies, the Nurses’ Health Study and the Health Professionals Follow-up Study in the U.S., indicated that a daily increase to three servings of fish was associated with a 7% lower risk of subjective cognitive decline (33). A similar finding was also reported in China. A cohort study based on the CHNS demonstrated that daily fish consumption of at least 100 g was related to better cognitive performance among adults aged 65 years and above in China, suggesting a potential protective role of fish in cognitive health (6). The specific mechanisms underlying the protective benefits of fish consumption on cognitive health may be related to the rich content of omega-3 polyunsaturated fatty acids in fish, including alpha-linolenic acid (ALA), eicosapentaenoic acid (EPA), and DHA. In particular, DHA, which is widely distributed in neural tissues, is a critical component of synaptic membranes. It plays a beneficial role in regulating neurogenesis, neuronal membrane permeability, synaptic plasticity, and neurotransmission (34). DHA has the capacity to mitigate both inflammation and oxidative stress within the central nervous system, thereby protecting neurons and inhibiting apoptosis (35, 36).

Our research has several strengths. For example, the CHNS data used are widely representative of mainland China. This study is also the first to demonstrate that adherence to the DGC differentially correlates with specific cognitive subdomains. The association between fish consumption and cognition in older adults was explored after controlling for other food components. Several limitations should also be noted. First, although this prospective study excluded individuals with mental/psychiatric disorders, intellectual disabilities, or neurological diseases, the absence of baseline cognitive assessments and relatively short follow-up period precluded definitive causal inferences. Second, while the three 24-h dietary recalls represent a validated assessment method, they may inadequately reflect long-term dietary patterns, potentially attenuating true diet-cognition associations. Additionally, although baseline dietary data are commonly used in nutritional epidemiology, this approach cannot account for dietary changes during follow-up. Repeated dietary measurements in future studies could mitigate this limitation. Finally, compared to those lost to follow-up, retained participants were younger, had lower diabetes prevalence, and higher energy intake, which may affect the generalizability of our findings.

## Conclusion

We observed that adherence to the DGC was linked to global cognitive performance as well as specific cognitive subdomains such as processing speed and attention/executive function among older Chinese adults. Considering both the potential significance and the constraints of this study, future research should verify these results using larger prospective cohort studies.

## Data Availability

Publicly available datasets were analyzed in this study. This data can be found here: https://www.cpc.unc.edu/projects/china.

## Footnotes

^1^ https://www.cpc.unc.edu/projects/china
