# Supplementary material for: Dietary adherence and cognitive performance in older adults: insights from a nationwide survey in China
Source: Front Nutr. 2025 Jul 30;12:1605016. doi: 10.3389/fnut.2025.1605016 (PMC12343236; doi:10.3389/fnut.2025.1605016)
Supplement: Supplementary file 1 [file Table_1.docx]

**Table S1. Comparison of baseline characteristics between participants who lost to follow-up and participants who completed follow-up.**

| Characteristics | CHNS participants who lost to follow-up | CHNS participants who completed follow-up | *P*^1^ |
| --- | --- | --- | --- |
| Age (years) | 70.9 ± 8.3 | 67.8 ± 6.4 | $<$ 0.001 |
| Gender, % |  |  | 0.459 |
| Male | 49.0 | 47.6 |  |
| Female | 51.0 | 52.4 |  |
| Nationality, % |  |  | 0.006 |
| Han | 91.8 | 88.7 |  |
| Others | 8.2 | 11.3 |  |
| Location, % |  |  | $<$ 0.001 |
| Urban site | 50.5 | 39.3 |  |
| Rural site | 49.5 | 60.7 |  |
| Education, % |  |  | 0.126 |
| Middle school and below | 80.9 | 83.0 |  |
| High school and above | 19.1 | 17.0 |  |
| Income, % |  |  | 0.053 |
| Low | 32.1 | 33.8 |  |
| Moderate | 31.6 | 34.1 |  |
| High | 36.3 | 32.1 |  |
| Energy intake (kcal/day) | 1764.4 ± 639.3 | 1866.95 ± 662.2 | $<$ 0.001 |
| Leisure sedentary time (hour/week) | 19.2 ± 15.4 | 18.9 ± 15.0 | 0.564 |
| Smoker, % |  |  | 0.763 |
| Yes | 25.9 | 25.4 |  |
| No | 74.1 | 74.6 |  |
| Drinker, % |  |  | 0.068 |
| Yes | 25.1 | 28.1 |  |
| No | 74.9 | 71.9 |  |
| Body mass index (kg/m^2^) | 23.6 ± 5.9 | 23.9 ± 5.3 | 0.072 |
| Hypertension, % |  |  | 0.826 |
| Yes | 49.3 | 48.9 |  |
| No | 50.7 | 51.1 |  |
| Diabetes, % |  |  | 0.001 |
| Yes | 10.4 | 7.2 |  |
| No | 89.6 | 92.8 |  |

^1^*P*-values were calculated using chi-square tests for categorical variables and F-tests for continuous variables.

**Table S2. Components and scoring standards of the CHEI.**

| Component | Maximum points | Standard for maximum score | Standard for minimum score of zero |
| --- | --- | --- | --- |
| Adequacy |  |  |  |
| Total grains | 5 | $\geq$2.5 SP/1000 kcal | 0 |
| Whole grains and mixed beans | 5 | $\geq$0.6 SP/1000 kcal | 0 |
| Tubers | 5 | $\geq$0.3 SP/1000 kcal | 0 |
| Total vegetables | 5 | $\geq$1.9 SP/1000 kcal | 0 |
| Dark vegetables | 5 | $\geq$ 0.9 SP/1000 kcal | 0 |
| Fruits | 10 | $\geq$ 1.1 SP/1000 kcal | 0 |
| Dairy | 5 | $\geq$0.5 SP/1000 kcal | 0 |
| Soybeans | 5 | $\geq$0.4 SP/1000 kcal | 0 |
| Fish and seafood | 5 | $\geq$0.6 SP/1000 kcal | 0 |
| Poultry | 5 | $\geq$ 0.3 SP/1000 kcal | 0 |
| Eggs | 5 | $\geq$0.5 SP/1000 kcal | 0 |
| Seeds and nuts | 5 | $\geq$0.4 SP/1000 kcal | 0 |
| Moderation |  |  |  |
| Red meat | 5 | $\leq$0.4 SP/1000 kcal | $\geq$3.5 SP/1000 kcal |
| Cooking oil | 10 | $\leq$ 15.6 g/1000 kcal | $\geq$32.6 g/1000 kcal |
| Sodium | 10 | $\leq$ 1000 mg/1000 kcal | $\geq$3608 mg/1000 kcal |
| Added sugar | 5 | $\leq$ 10% of energy | $\geq$20% of energy |
| Alcohol | 5 | $\leq$25 g (men)/15 g (women) | $\geq$ 60 g (men)/40 g (women) |

**Table S3. Sensitivity analysis on the associations between the CHEI and poor cognitive performance when excluding participants with diabetes** **(n = 2026)^1^.**

| Models | CHEI | | | |  |  |
| --- | --- | --- | --- | --- | --- | --- |
|  | Q1^2^ | Q2 | Q3 | Q4 | *P*_trend_^3^ | 1 SD increase |
| Model 1^4^ | 1 (Reference) | 0.82 (0.61, 1.11) | 0.68 (0.50, 0.92)^*^ | 0.34 (0.24, 0.48)^***^ | $<$ 0.001 | 0.69 (0.62, 0.78)^***^ |
| Model 2 | 1 (Reference) | 0.86 (0.64, 1.16) | 0.73 (0.54, 1.00)^*^ | 0.44 (0.30, 0.63)^***^ | $<$ 0.001 | 0.76 (0.67, 0.86)^***^ |
| Model 3 | 1 (Reference) | 0.86 (0.63, 1.16) | 0.79 (0.58, 1.08) | 0.55 (0.38, 0.80)^**^ | $<$ 0.01 | 0.83 (0.73, 0.94)^**^ |

^1^CHEI: Chinese Healthy Eating Index; ^***^*P* $<$ 0.001, ^**^*P* $<$ 0.01, ^*^*P* $<$ 0.05.

^2^Q: Quartile, Q1 represents the unhealthiest diet quality, Q4 represents the healthiest diet quality.

^3^*P*_trend_: Test for trend based on a variable containing the median value for each quartile.

^4^Model 1: adjusted for age, gender and nationality; Model 2: Model 1+location, energy intake, drinking status, smoking status, and leisure sedentary time; Model 3: Model 2+education, income, BMI, and hypertension.

**Table S4. Sensitivity analysis on the associations between the CHEI and poor cognitive performance when excluding participants with overweight or obesity (n = 1200)^1^.**

| Models | CHEI | | | |  |  |
| --- | --- | --- | --- | --- | --- | --- |
|  | Q1^2^ | Q2 | Q3 | Q4 | *P*_trend_^3^ | 1 SD increase |
| Model 1^4^ | 1 (Reference) | 0.70 (0.48, 1.03) | 0.77 (0.53, 1.12) | 0.33 (0.21, 0.51)^***^ | $<$ 0.001 | 0.70 (0.60, 0.81)^***^ |
| Model 2 | 1 (Reference) | 0.73 (0.50, 1.08) | 0.82 (0.56, 1.20) | 0.45 (0.28, 0.70)^**^ | $<$ 0.01 | 0.78 (0.66, 0.91)^**^ |
| Model 3 | 1 (Reference) | 0.74 (0.50, 1.09) | 0.88 (0.60, 1.29) | 0.54 (0.34, 0.87)^*^ | $<$ 0.05 | 0.84 (0.71, 0.99)^*^ |

^1^CHEI: Chinese Healthy Eating Index; ^***^*P* $<$ 0.001, ^**^*P* $<$ 0.01, ^*^*P* $<$ 0.05.

^2^Q: Quartile, Q1 represents the unhealthiest diet quality, Q4 represents the healthiest diet quality.

^3^*P*_trend_: Test for trend based on a variable containing the median value for each quartile.

^4^Model 1: adjusted for age, gender and nationality; Model 2: Model 1+location, energy intake, drinking status, smoking status, and leisure sedentary time; Model 3: Model 2+education, income, BMI, hypertension, and diabetes.

**Table S5. Sensitivity analysis on the associations between the CHEI and composite z-score of cognitive performance (n =2174)^1^.**

| Models | CHEI | | | |  |  |
| --- | --- | --- | --- | --- | --- | --- |
|  | Q1^2^ | Q2 | Q3 | Q4 | *P*_trend_^3^ | 1 SD increase |
| Model 1^4^ | 0 (Reference) | 0.24 (-0.02, 0.49) | 0.44 (0.19, 0.69)^**^ | 0.95 (0.69, 1.20)^***^ | $<$ 0.001 | 0.35 (0.26, 0.44)^***^ |
| Model 2 | 0 (Reference) | 0.20 (-0.05, 0.45) | 0.37 (0.12, 0.62)^**^ | 0.70 (0.44, 0.96)^***^ | $<$ 0.001 | 0.25 (0.16, 0.35)^***^ |
| Model 3 | 0 (Reference) | 0.19 (-0.05, 0.44) | 0.28 (0.03, 0.52)^*^ | 0.46 (0.19, 0.72)^**^ | $<$ 0.01 | 0.14 (0.05, 0.24)^**^ |

^1^CHEI: Chinese Healthy Eating Index; ^***^*P* $<$ 0.001, ^**^*P* $<$ 0.01, ^*^*P* $<$ 0.05.

^2^Q: Quartile, Q1 represents the unhealthiest diet quality, Q4 represents the healthiest diet quality.

^3^*P*_trend_: Test for trend based on a variable containing the median value for each quartile.

^4^Model 1: adjusted for age, gender and nationality; Model 2: Model 1+location, energy intake, drinking status, smoking status, and leisure sedentary time; Model 3: Model 2+education, income, BMI, hypertension, and diabetes.

**
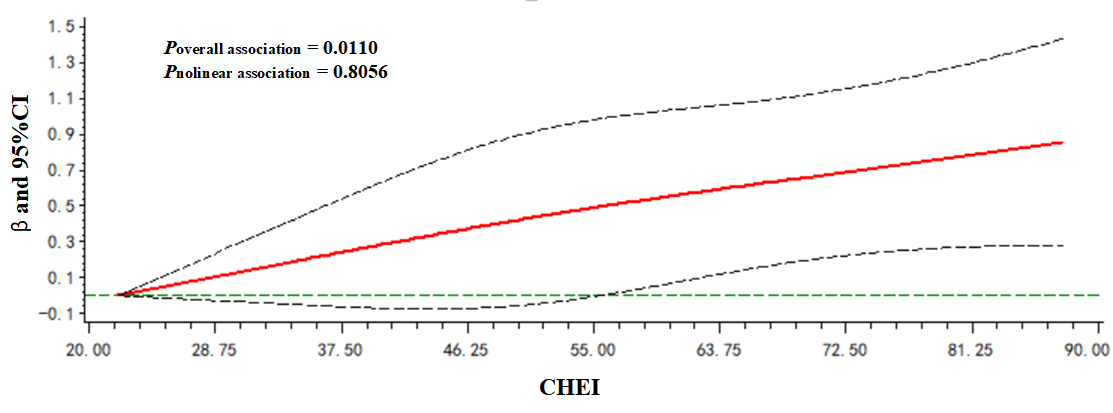
**

**Figure S1.** Dose-response relationship between the CHEI and composite z-score of cognitive performance. Models were adjusted for age, gender, nationality, location, energy intake, drinking status, smoking status, leisure sedentary time, education level, income, BMI, hypertension, and diabetes.
